# Interesting Case of Supposed Carotid Aneurism, Where the Artery Was Tied beyond the Tumour

**Published:** 1830-01-01

**Authors:** 


### CASE OF CAROTID ANEURISM WHERE THE ARTERY WAS TAKEN UP ABOVE THE TUMOUR.

This case, for which we are indebted to the politeness of Sir Jas. M'Grigor, is of so interesting a character that we hasten to publish it. It is one of a thousand instances of that zeal for science which honourably distinguishes our brethren of the medical department of the army and navy.

The subject of this very formidable disease was a free black, aged about 40 years, a creole of this colony, and was employed as a gent d'armes; tall, of a spare habit of body, and rather given to intemperance. Was admitted into the Civil Government Hospital (of which I am surgeon in charge) on the 20th February of the present year, for an aneurismal tumour the size of a pullet's egg, situated immediately above the sternal portion of the left clavicle, and so close to that bone, that it seemed to emerge from behind it, or rather from within the cavity of the chest; which rendered the taking up of the common carotid artery below the aneurism absolutely impracticable.

The poor man had an almost constant tickling cough, with severe pain of the trachea; copious frothy mucous expectoration; great anxiety of countenance; hoarseness of voice; disturbed sleep; and was rather emaciated from constant watching.

The account he gave of his case was, that he caught cold by sleeping exposed in the night air about a fortnight prior to his admission into hospital, at which period he first noticed the tumour. As there was considerable derangement of the digestive organs, the requisite cathartic medicines were given to restore their functions, and expectorants and anodynes for the cough and general irritation.

21st. Has headache with severe fixed pain of the left temple; considerable fever; pulse 76, tense, and full, but irregular; the tumour increasing and pulsating strongly; has been three times copiously purged. V.S. ad ℥x. mist. salinæ simp. ℥j. cum tart. ant. gr.¼, 3tiis horis. Vespere, The bleeding produced tendency to fainting, and has relieved the head-ache and fever, but the pain of temple and other symptoms continue. From this period the tumour went on increasing rapidly until 9th March, when it had acquired an alarming size, the base occupying the space of two-thirds of the sternal portion of the clavicle, and ascending nearly four inches upwards to the angle of the jaw, so that the volume of the tumour limited exceedingly the space for taking up the artery above it—the attempting of which I was induced to undertake from having read Mr. Wardrop's successful case in the 9th volume of the Lancet, folios 479 to 485. Seeing that no time was to be lost, as the tumour might soon burst, or the patient be suffocated by its pressure on the trachea, and that its rapidly increasing size would soon so far diminish the space for operating as to render an operation impossible, I requested a consultation of the principal medical officers of Port Louis, as well French as English, who all unanimously concurred with me in opinion that the taking up of the artery, ultra tumorem, was an advisable measure. I immediately commenced the operation in the presence of numerous medical spectators, aided by my friends Dr. Ingham, Surgeon, 29th Regiment, and Dr. Shanks Assist. Surgeon, 82nd Regiment, and Act. Chief of the Civil Medical Department. It may be observed here, that I was badly supplied with instruments, the want of which was however compensated by my able assistants. The operation was performed in a similar manner to that described by Mr. Wardrop, and consisted in making an incision of an inch and a quarter long through the integuments and platysma myoides muscle, by which the inner edge of the sterno cleido mastoideus was brought into view, greatly thrown inward and forward out of its natural position. This being drawn aside by a retractor, the incision was continued on its inner side in the direction of the carotid artery with every possible caution to avoid the superficial veins, one of which, of considerable size, crossed the neck, limiting very much my space for operating. The after part of the operation was attempted with a silver knife, as directed by Mr. Wardrop, but finding that instrument too clumsy, and depending on the steadiness of my hand, I removed the cellular substance, and exposed the sheath of the vessels, by dissecting with the forceps and scalpel, occasionally using the handle of the latter, when I found it necessary. This dissection exposed the descendens noni running on the front of the sheath of the vessels, the bifurcation of the artery, the external jugular vein at the upper angle of the incision; and a vein the size of a large crow-quill crossing the artery at the lower angle, and immediately above the omo-hyoideus muscle, which limited the space for taking up the artery to little more than half an inch. The sheath of the vessel was now slit open, when the artery, vein, and par vagum nerve, were seen in their natural situations.

The patient who had been very restless during the whole of the operation, suddenly raised himself up, so that I was compelled to seek again for the sheath, and being unable to find the first opening, I was obliged to make a second one. An attempt was now made to pass a rude crooked aneurismal needle armed with a double ligature around the artery, in which I was foiled by the restless state of the patient. A second attempt proved more successful, as it passed with facility. On the ligature being laid hold of, the needle was withdrawn and on one ligature being secured the other was removed and the wound brought in contact by a simple suture and strap of adhesive plaster. The patient being now faint, a little wine and water was given and he was put to bed. It is remarkable that scarcely a drop of blood was lost during the operation (which was performed in about 25 minutes) except what escaped from the vessels of the integuments and platysma myoides, which did not exceed a tea-spoonful.

March 10th. The patient suffered very much from dyspnœa, cough, considerable and increased frothy mucous expectoration, and difficult deglutition for several hours after the operation, but which symptoms are now much abated. Pain of temple entirely gone; slept none in the night, for which he cannot account; pulse 80, soft and full; tongue white, belly slow, the pulsation of tumour less distinct, and he feels in every respect much relieved. Ol. Ricini, ℥j. Mist. Mucil. pro tusse. Vespere, well purged; Haust. Tinct. Opii. gtt. xxv.

March 11th. Passed a tolerably good night; has less cough and irritation of the trachea; suffers but little from the wound, or tumour, in which there is still pulsation, but less distinct than prior to the operation; complains of fixed pain of left scapula; pulse at the wrist 88, soft tolerably full and irregularly intermittent; skin natural; but little thirst. Mist. Salinæ Simp. ℥j. Tr. Digitalis, gtt. viij. 3tiis horis. Potus Lemonade. Vespere, continues to go on well—pulse 72, no disposition to sleep. Haust. Tr. Opii, gtt. xxx.

March 12th. Passed a good night; tumour much decreased in size; and says that the pulsation has entirely subsided, neither is it to be felt. Complains chiefly of slight difficulty of deglutition (but less so than at any period subsequent to the operation) with a fulness at the epigastrium and flatulent eructation. Voice more clear and distinct; pulse 78, soft and tolerably full, but still intermitting; no motion of bowels. Haust. cathart.; continue mixture and potus.

March 13th. Has had but one scanty stool; slept none until an anodyne was given at midnight, after which he slept well till five o'clock. Pulse 80, soft, tolerably full, and intermitting at longer intervals. The dressings being removed the wound presented no appearance of union.

14th. The aneurismal tumour is reduced to one half its original size, and does not pulsate when the patient is sitting up in bed. In the recumbent posture, however, an indistinct pulsation becomes perceptible. The patient is not sensible of any pulsatory movement in the tumour, but the wound has been painful and caused a restless night. Bitter tonic mixt. ℥j. thrice a day; pil. hyd. gr. v. h. s.

15th. A tolerably good night; bowels still confined; uneasiness of chest and epigastrium; pulse 70, soft, full, and more regular; cough diminished; expectorates with difficulty; wound less painful. Ol. ricini, ℥j. statim. Mist. mucilag. pro tusse.

16th. Bowels freely opened; all the symptoms less urgent; dressings removed; wound healthy; tumour diminishing. Contin. mist. mucilag.

17th. Tumour continues to decrease; all the symptoms less urgent.

18th. Tumour lessened; distinct pulsation perceptible at a small point on the humeral edge of the aneurism, indicative of approaching rupture of the sac; uneasy sensation of left side of chest; deglutition and cough much relieved; bowels confined. Ol. ricini, ℥j.

19th. Well purged; passed a good night; wound dressed and is nearly healed; ligature still attached; aneurismal tumour as yesterday; pulsation at the point mentioned less distinct; general feelings much improved. Infus. quassiæ, ℥j. ter in die. Pil. hyd. gr. v. h. s.

20th. Goes on well. In the evening slight hæmorrhage from the wound, which, excepting where the ligature comes out, is entirely healed. Pulse mnch excited; general agitation and dread of approaching death. Pulsation at the point mentioned on 18th more distinct, but no where else over the tumour. Haust. ex tinct. digital. p. gtt. xx. Tt. camph. c. gtt. xl.

21st. An indifferent night; no return of hæmorrhage; pulsation at the point specified still distinct; pulse irregularly intermitting; belly confined. Ol. ricini, ℥j.

22d. Belly relieved; considerable return of hæmorrhage at half past 10 o'clock last night, followed by chills and total cessation of pulsation at the point alluded to, but which has returned since 5 o'clock this morning; pulse irregularly intermitting. At 9, a.m. considerable bleeding from the wound; and at 2 and 4, p.m. bleeding recurred, but on every occasion was easily commanded.

23d. A good night; no renewal of hæmorrhage; aneurismal tumour more distended, pulsating considerably; pulse irregularly intermitting; belly confined. Pil. hyd. gr. v. h. s.

March 24th. Slept none; bowels unopened; no bleeding; pulse 80, soft, full, and more regular. Ol. ricini, ℥j.

25th. Four copious stools; no hæmorrhage; pulse 80, soft, full, and regular; a good night, pulsation of aneurism lessened.

26th. Continues as yesterday; dressed the wound, which discharged pus slightly tinged with blood. Aneurism nearly of same size; no pulsation perceptible. Being watchful in the evening got an anodyne.

27th. Bowels torpid; otherwise as above; ol. ricini, ℥j.

28th. Twice purged; wound dressed; ligature came away with the dressings. Aneurismal tumour appears enlarged; deglutition again difficult. Mist. mucilag. ut antea.

29th. A restless night; belly confined; tongue foul; ol. ricini, ℥j.—From this period until 3d of April, no change of importance occurred. At this date, a small abscess had formed in the course of the cicatrix, which discharged itself through the small opening left by the ligature, but by the 5th the discharge had ceased, and the opening specified finally closed.

April 6th. Palpitation of heart and throbbing in the aneurismal tumour in the night; bowels confined; haust. cathart.

7th. Purged freely; tumour painful in the night, but not pulsating. Pulse 84, soft, full, and regular.

May 28th. Since last report the general symptoms have been unimportant; the tumour gradually enlarged and threatened to suppurate, and the pointing prominence noticed on 18th March was so thin, as to cause apprehension of its bursting momentarily. The next day (29th) it gave way, discharging about eight ounces of fetid chocolate-coloured fluid. Compresses and bandage were applied to prevent the apprehended hæmorrhagy. On the 30th, these dressings, soaked with fetid discharge, were removed; there being no sanguineous effusion, and perceiving the opening of the pointed tumour to be insufficient to give exit to the corrupted aneurismal blood, I ventured to enlarge it. The incision being made, from 6 to 8 ozs. of matter similar to the above, mixed with coagula, escaped. I introduced my finger and removed a considerable quantity of coagula and tenacious lymph. In the act of moving my finger for this purpose I felt the artery, below the seat of the ligature, without pulsation, the trachea pushed considerably to the right side, the anterior surface of the cervical vertebræ, and the muscles sterno-hyoideus and thyroideus as if dissected; the sterno cleido mastoideus rounded, and as if knotty, admitting the finger to pass round it. After clearing out the sac a dossil of lint was introduced, and adhesive straps with bandage applied. These dressings being removed on the following day, the lint was found covered with pus, but no discharge from the wound, which looked tolerably well. The swelling had very considerably subsided, the patient had passed a good night, breathed easier, coughed and expectorated less, and the pulse, from 106, had fallen to 80. From this period the countenance and general condition of the patient improved; and every day's visit gave additional reason to hope for his recovery. The great size of the tumour may in part be accounted for by the decomposition of blood and disengagement of gas. The fetor of the matter was such, that I could not remove it from my fingers for two days.

At the present period (8th June) the patient begins to walk out of doors, there is no discharge from the wound, which is on the eve of healing: all tumour has entirely disappeared from the neck, and whatsoever fate be in reserve for the patient, the aneurism at least seems to be cured.

This case, as may naturally be supposed, has excited considerable interest in this colony. All the professional gentlemen who favoured me with their presence at the operation have occasionally visited the patient since it was performed. In a future communication I shall have the honor of informing the profession of the further history of the patient.

A. Montgomery,
*Surg. R.N. & Surg. Civil Govmt. Hosp. in charge.*
*Mauritius, June 9th,* 1829.

